# Assessing the Ecological Risk of Polycyclic Aromatic Hydrocarbons in Sediments at Langkawi Island, Malaysia

**DOI:** 10.1155/2013/858309

**Published:** 2013-09-17

**Authors:** Essam Nasher, Lee Yook Heng, Zuriati Zakaria, Salmijah Surif

**Affiliations:** ^1^Faculty of Science and Technology, Universiti Kebangsaan Malaysia, 43600 Bangi, Selangor, Malaysia; ^2^South-East Asia Disaster Prevention Research Institute (SEADPRI), Universiti Kebangsaan Malaysia, 43600 Bangi, Selangor, Malaysia; ^3^Environment Engineering and Green Technology, Malaysia Japan International Institute of Technology, University Technologi Malaysia, 54100 Kuala Lumpur, Selangor, Malaysia

## Abstract

Tourism-related activities such as the heavy use of boats for transportation are a significant source of petroleum hydrocarbons that may harm the ecosystem of Langkawi Island. The contamination and toxicity levels of polycyclic aromatic hydrocarbon (PAH) in the sediments of Langkawi were evaluated using sediment quality guidelines (SQGs) and toxic equivalent factors. Ten samples were collected from jetties and fish farms around the island in December 2010. A gas chromatography/flame ionization detector (GC/FID) was used to analyse the 18 PAHs. The concentration of total PAHs was found to range from 869 ± 00 to 1637 ± 20 ng g^−1^ with a mean concentration of 1167.00 ± 24 ng g^−1^, lower than the SQG effects range-low (3442 ng g^−1^). The results indicated that PAHs may not cause acute biological damage. Diagnostic ratios and principal component analysis suggested that the PAHs were likely to originate from pyrogenic and petrogenic sources. The toxic equivalent concentrations of the PAHs ranged from 76.3 to 177 ng TEQ/g d.w., which is lower compared to similar studies. The results of mean effects range-median quotient of the PAHs were lower than 0.1, which indicate an 11% probability of toxicity effect. Hence, the sampling sites were determined to be the low-priority sites.

## 1. Introduction

Langkawi Archipelago in the Straits of Malacca, northwest of Peninsular Malaysia, consists of 104 islands, the largest and most exploited of which is Langkawi Island with an area of 478.5 km^2^. Targeted for ecotourism, Langkawi is protected from engaging in heavy industrial activities [1]. In 2006, the island was declared a National Geopark, and in the following year, it became a UNESCO Global Geopark [2]. In 2010 alone, Langkawi was visited by 2.4 million tourists [2]. From approximately 40,000 in 1991, the local population swelled significantly to almost 100,000 in 2010 [1], mainly to cater to the increasing number of tourism-related activities. Unfortunately, the fragile ecosystem of Langkawi Island is also increasingly being threatened by these tourism activities. Among the main attractions of Langkawi are its unique geological formations accessible only by boats. Many fish farms cater to the fresh seafood restaurants that are appearing around the islands. Boating activities, which are an important tourism component in Langkawi, significantly increase petroleum and diesel pollution in the water around the island. One of the most significant polluting components of petroleum is polycyclic aromatic hydrocarbons (PAHs).

PAHs are a group of over 100 different compounds with fused benzene rings [3]. Sixteen PAHs compounds are identified as priority pollutants due to their toxic, mutagenic, and carcinogenic characteristics [4]. PAHs in the environment can result from petroleum and petroleum products (petrogenic) or from the incomplete or inefficient combustion of diesel fuel, engine oil, wood, coal, biomass of forest, grass fires, waste incinerators, and fossil fuels, all of which are commonly used in industrial operations and power plants (pyrogenic) [5, 6]. PAHs are also widely used in commercial products such as intermediaries in pharmaceuticals, agricultural products, photographic products, thermosetting plastics, and lubricating materials, products that may end up contaminating the environment.

In the marine environment pollution from PAHs can be due to natural seepage or land-based sources, river discharges, urban runoffs, refineries, and other industrial wastewaters [7]. Sea-based sources, on the other hand, are from two-stroke vessel discharge, nontank vessel spills, operational discharges, gross atmospheric deposition, and aircraft dumping [8]. These various sources of PAHs can be differentiated by their diagnostic ratio: anthracene to anthracene + phenanthrene (Ant/Ant+Phe) ratio of >0.1 indicates that the PAHs are pyrogenic, whereas a ratio of <0.1 shows that they are petrogenic in origin [9]. 

The ecological risk of PAHs in sediment is evaluated based on the effects range-low (ERL) and effects range-median (ERM) values of the effects-based sediment guideline [10]. These two values establish three concentration ranges for PAHs. At concentrations <ERL, biological effects rarely occur; at concentrations ≥ERL ≤ERM biological effects occasionally occur; and at concentrations >ERM, negative biological effects frequently occur [11]. The risk assessment of PAHs in the aquatic sediment of certain regions has been reported in several studies [12–16]. 

To date, no investigation has been conducted on the impact of the tourism sector in Langkawi. In this paper, we report the distribution, composition, sources, and pollution level of PAHs in the sediments of jetties and selected fish farms within areas around Langkawi Island, which is the focal point of the marine tourism industry. We also evaluate potential biological toxicity and its impact on the local ecosystem of the area. 

## 2. Materials and Methods

### 2.1. Chemicals and Reagent

A standard mixture of PAHs consisting of naphthalene (Nap), 1-methylnaphthalene (1MNap), 2-methylnaphthalene (2MNap), acenaphthylene (Acy), acenaphthene (Ace), fluorene (Fl), phenanthrene (Phe), anthracene (Ant), fluoranthene (Flu), pyrene (Pyr), benz[a]anthracene (BaA), chrysene (Chr), benzo[b]fluoranthene (BbF), benzo[k]fluoranthene (BkF), benzo[a]pyrene (BaP), indeno[1,2,3-cd]pyrene (InP), dibenzo[ah]anthracene (DBA), and benzo[ghi]perylene (BgP) was purchased from Restek Corporation, USA. The standard mixture was diluted with hexane to prepare the five calibration standard mixtures. The *p*-terphenyl-d14 (*p*-Ter) (Supelco, USA) was used as the surrogate internal standard. Dichloromethane (DCM), n-hexane, pentane, acetone, and cyclohexane were all of chromatographic grade. 

### 2.2. Study Area

**Figure 1:**
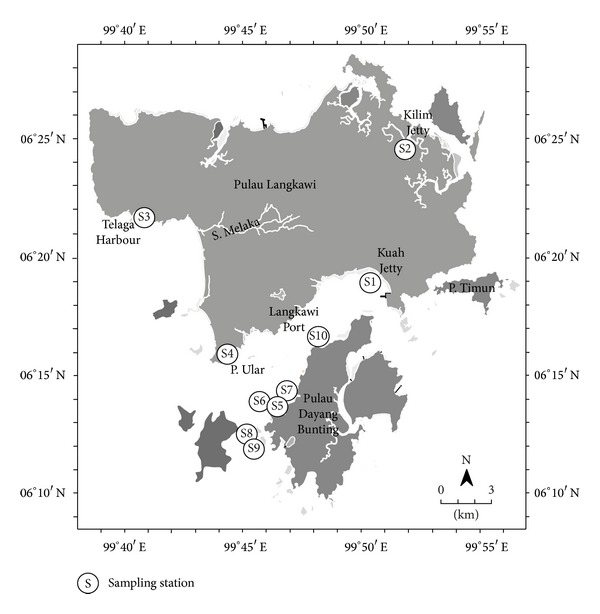
Map showing the ten sampling stations (S1 to S10) around Langkawi Island, Malaysia.

The sediment was sampled in December 2010.  Figure 1 and Table 1 show the ten sampling stations around Langkawi Island and their associated water depths, respectively. The sampling stations include Telaga Harbour, Kilim Jetty, Porto Malai Jetty, Kuah Jetty, and six stations in fish farms (I, II, and III) located approximately 10 km to 20 km from Porto Malai Jetty. These sampling stations were chosen based on their unique activities: Telaga Harbor is a terminal jetty for sailing boats and yachts, while Kilim Jetty and Porto Malai Jetty are both starting points for ecotourism activities. Kuah Jetty is the main terminal for ferries from mainland Kuala Kedah and Penang, as well as Thailand and Singapore. Fish farms (I, II, and III) are chosen to represent the many fish farms and finfish aquacultures in the coastal water surrounding Langkawi Island that are frequented by tourists.

### 2.3. Sample Collection

Stainless steel Van Veen grab sampler was used to collect surface sediment. The surface layer of the upper five centimeters was carefully taken using a stainless steel spatula previously cleaned with n-hexane. The surface sediments were wrapped in aluminium foil, kept in plastic bags, and stored in a dark place under −4°C until further analysis. A sediment sample (100 g) was taken from each station, placed in a precleaned glass bottle, and weighed before drying in a freeze-dryer (Labconco Lyph Lock 6, Model: 77530-00). Duplicate samples from each station were used for analysis.

### 2.4. Grain Size and Organic Matter Analysis

Wet sediments were weighed accurately and dried in the freeze-dryer (Labconco Lyph Lock 6, Model: 77530-00) for 72 h. The sediments were carefully dispersed by mortar to keep the nature of each grain size. They were then placed in vibratory sieve shaker (AS 300) containing a series of sieves (2, 1, 0.5, 0.25, 0.125, 0.063, and 0.045 mm) and agitated for 10 mins. The sediments in each sieve were transferred using a metal brush to a preweighed tray and was weighed again. Each grain size fraction was taken as a percentage of the total mass of the whole sediment. Sediment grain size was classified as gravel (>1 mm), sand (1 to 0.063 mm), or silt and clay (<0.063 mm). Sediments that contained medium, fine sand and silt with sizes ≤0.25 mm were used for PAHs analysis.

The OM content in dry sediment was measured using the method explained by Briggs [17]. Dry sediment (2 g) from each station was placed in a clean preweighed porcelain dish and heated in a furnace at 550°C for 6 h. The percentage of OM was calculated based on the mass ratio of sediment weight in the porcelain dish before and after heating.

### 2.5. Total Organic Carbon

The TOC content in sediment was measured as described by Dahle et al. [18]. A sediment sample with fixed weight was acidified using concentrated HCl to bring the pH down to ≤2 and remove the inorganic carbons. The sample was then dried in an oven at 50°C for 2 days. The TOC content was analysed through the high temperature combustion method using a CHNS (O) Analyzer (Thermo Finnigan, Italy). 

### 2.6. Chemical Analysis

Hydrocarbon pollutants were extracted from the sediment sample according to the USEPA method 3540C [19]. The clean-up process followed the procedures described in the EPA method 3630C [20]. Briefly, 10 g dry sediment was spiked with the surrogate standard *p*-terphenyl-d14 (2 *μ*g mL^−1^) and extracted into a Soxhlet apparatus using 200 mL GC-grade acetone: DCM (v : v 1 : 1) for 10 h. The combined extract was mixed with activated copper sulphate granules and left overnight to remove any sulphur contaminants. Next, the extract was passed through a glass column that contained glass wool and then concentrated to 3 mL using a rotary evaporator (EYELA, Japan, model: N-1001S-W). Cyclohexane (10 mL) was added as an exchange solvent, and the extract was concentrated to 2 mL using a rotary evaporator. The extract was again passed through a glass column containing 5 g activated silica gel (previously activated by heating at 200°C for 16 h before use) and 1 g of anhydrous Na_2_SO_4_. Afterward, the PAH fraction was eluted using a 30 mL mixture of DCM : pentane (2 : 3, v/v) and then concentrated to 2 mL using a rotary evaporator. Hexane (10 mL) was added as an exchange solvent [20] and evaporated down to 2 mL. Finally, the extract was reduced to 1 mL under a gentle stream of nitrogen gas. All sample extracts were kept in amber glass vials at −4°C until they were analysed within a week.

### 2.7. Instrumental Analysis

Extract (1 *μ*L) was injected into gas chromatography (Agilent 6890 technologies, USA) equipped with flame ionization detector (FID) and a fused silica TR-5MS capillary column (30 m × 0.25 mm i.d.) with film thickness of 0.25 *μ*m (Thermo Fisher, USA). High purity helium (99.9%) was used as a carrier gas, makeup gas, and purge gas at flow rates of 1.0, 45, and 30.0 mL/min, respectively. The flow rates for the FID were 450 mL/min and 45 mL/min for air and hydrogen, respectively. The gas chromatograph was operated in splitless mode, and separation was conducted with the oven temperature programmed as follows: initial setting at 80°C (1 min hold), ramped to 180°C at 10°C/min (for 2 min), and finally to 320°C at 5°C/min (10 min hold). The injector was held at 250°C and the FID maintained at 350°C. Agilent Chemstation software was used to obtain the chromatogram and for data calculations. An external standard calibration comprising of 18 PAH standards was used to determine the identity and quantity of each component peak in sample chromatogram.

### 2.8. Quality Assurance and Quality Control (QA/QC)

Replicate samples were analysed for all samples collected from each station. Reagent blank and recovery procedures were analysed simultaneously for every five samples. The reagent blank containing the surrogate standard and solvent was analysed to evaluate the interference and contamination of the solvents, reagents, and glassware used. The accuracy of the analytical procedure was examined by recovering the PAHs in the standard reference material (SRM) 1941b (marine sediment) obtained from the National Institute of Standards and Technology (NIST, USA). 

The extraction, clean-up procedures, and setting up of instrumental system were examined by spiking each real sample and reagent blank with a surrogate internal standard (*p*-terphenyl-d14) of a known concentration. Average recovery of the 18 PAHs and *p*-terphenyl-d14 ranged from 65 to 137% (Table 2), which met the 70–130% acceptance criteria of the EPA method [21]. The instrument limit of detection of individual PAHs was estimated to be 3∗*S* where *S* is the standard deviation of eight replicate analyses of blanks [22]. The method detection limit (MDL) was calculated to be 10∗*S*. The MDL values of the individual PAHs ranged from 0.22 to 1.2 ng g^−1^ according to the analysis method with GC-FID. These values were within the acceptable range of EPA method [23]. 

The correlation coefficient (*r*), a measure of the “goodness of fit” of the regression line to the data, must be ≥0.99 to be acceptable for the regression equation. Five PAH mixture standards were run on the same day of the sample analysis to estimate the regression equations used to calculate the concentration of individual PAHs in the samples. All PAH regression equations obtained an *r* value of ≥0.99 which was acceptable according to the EPA method 8000B [21]. 

One-way ANOVA, Games-Howell, and posthoc multiple comparison tests were used to evaluate the significance of the differences between the total PAHs at the sampling stations using SPSS version 15 for Windows. Correlation Pearson analysis was carried out to test the relationship between individual PAHs in the sediment and between total PAHs, different grain size, and TOCs. Principal component analysis (PCA) was conducted to identify the source contributions of PAHs. 

## 3. Results and Discussion

### 3.1. Sediment Characteristics

Table 3 shows the TOC, OM content, and grain size of the sediment samples taken from 10 stations around Langkawi Island. TOC values ranged from 0.66% to 3.17%, and OM content was between 10.26% and 22.41% in dry weight. Kilim Jetty, which is located at the mouth of Kilim River and is covered by mangroves, recorded a higher TOC content than the studied locations. Kilim Jetty also has a higher percentage of OM compared to other jetties and harbour that have the same activities. OM includes the total amount of organic and inorganic carbon, nitrogen, and phosphorus [24]. The highest TOC and OM contents at Kilim Jetty are expected because the jetty is a mangrove area located at the mouth of Kilim River, which receive the discharges from biological productivity and high sedimentation rates [25]. The lowest TOC content (0.66%) was recorded at Telaga Harbour. The lower concentration of TOC in this station may be due to the low biological productivity. 

The OM contents (16.82–22.41%) in fish farms and in Kilim Jetty (Table 3) were higher in other stations (Kuah, Porto Malai jetties, and Telaga Harbour), indicating that feed wastage, fish excretion, and faecal productions from fish cages can contribute to the formation of loose and black flocculent under the mariculture cages which may then cause the accumulation of OM [26]. The high values of OM in fish farm sediment were also found in other sites. For example, the fish farms of Corse in France have a recorded OM value between 21% and 24% [27]. 

Based on grain size, the sediments of Langkawi Island were mostly sandy with sand content ranging from 89.72% at Porto Malai Jetty to 99.75% at fish farm III (Table 3). The clay content of these sediments was ≤1%. Significant relationships were found between sediment size (≤0.25 mm) and total organic carbon (TOC) (*r* = 0.68, *P* = 0.03) and between the size and organic matter (OM) content (*r* = 0.66, *P* = 0.05) of the sediment. PAHs are highly hydrophobic and usually accumulate in the sediment with high OM content [18].

### 3.2. Concentrations and Profile of PAHs

Table 4 shows the concentrations of PAHs in sediment from the four chosen jetties and six fish farm stations around Langkawi Island. The total PAH concentrations in the popular jetties for tourists, the Kuah Jetty, Kilim Jetty, Porto Malai Jetty, and Telaga Harbour, varied from 868 ± 85 ng g^−1^ d.w. in the Kilim Jetty to 1637 ± 190 ng g^−1^ d.w. in Telaga Harbour, with a mean of 1165 ± 235 ng g^−1^ d.w. Total PAHs in the sediment samples from the fish farm areas similarly recorded ranges of 922 ± 170 ng g^−1^ d.w. at fish farm I to 1432 ± 693 ng g^−1^ d.w. at fish farm III with a mean of 1143 ± 63.5 ng g^−1^ d.w. Sediment from Telaga Harbour sediment recorded the highest total PAHs concentration of 1637 ± 190 ng g^−1^ d.w., which could be due to the intense harbour activities of sailing boats and yachts in this popular terminal jetty. Additionally, the Telaga sampling site is located in the sheltered part of the water. The lowest concentration of total PAHs was found at Kilim Jetty at 868 ± 85 ng g^−1^ d.w, which is probably due to the dilution effect of the Kilim River. Kilim River crosses the forest and rural areas and is loaded with high OM that is then deposited at the jetty. The jetty has a depth of about 1 to 2 m and a water transparency of 0.80 cm. This discharge may leach the PAHs in the jetty sediment to the open sea. Moreover, biological activity may return a small amount of PAH to the water column, which contributes to reducing PAH level in the jetty [7]. The PAH pollution categories adopted by Baumard et al. [28] are as follows: low: 0–100 ng g^−1^, moderate: 100–1000 ng g^−1^, high: 1000–5000 ng g^−1^, and very high: >5000 ng g^−1^ d.w. On the basis of this classification, Langkawi Island can currently be considered moderately to heavily pollute with PAHs. Therefore, the prevailing PAH concentration at the jetties and fish farms frequently visited by tourists is probably the result of the fuel used in boats, passenger ferries, and buses, which are the important contributors of PAHs in this area. The leakage of petroleum and the unscrupulous disposal of engine oil from boats and ferries may also contribute significantly to the level of PAHs recorded here [29, 30]. 

To evaluate if the sediment contaminated by PAHs in Langkawi Island (868–1637 ng g^−1^ d.w.) will have a toxic effect [10], the total PAHs levels were also compared against effects-based guideline values such as ERL and ERM. The concentrations of total PAHs from all the studied stations were lower than the sediment quality guidelines (SQGs) for the ERL of 3442 ng g^−1^ and the ERM of 24290 ng g^−1^. These findings suggest that the sediments from these sampling locations are not toxic to the organisms within these areas.

Table 5 shows that the total PAH concentrations in the sediment of Langkawi Island were approximately lower by 1 to 2 orders of magnitude than the sediments investigated by other studies in Singapore Island, the Mediterranean coastal environment of Egypt [16], and the Naples harbour in southern Italy [32]. The levels recorded here are nearly similar to the levels present in the sediment of Jakarta Bay, Indonesia [33], Jiulong River Estuary and Western Xiamen Sea, China [34], and Tokyo Bay, Japan, [33]. However, total PAHs in the Langkawi sediment were approximately 1 order of magnitude higher than those detected in other countries, such as the marine sediments in Thailand [35], Estero de Urias Estuary, Mexico [36], the Gulf of Aden, Yemen [37], Southwest Taiwan [38], and in Hong Kong marine fish farms [26] (Table 5). Moreover, the 2006 annual report of the Malaysian Department of Environment ranked Langkawi Island as the third most polluted area by oil and grease among the 15 monitoring stations in the Peninsula of Malaysia. Langkawi also exceeded the water quality standard of oil and grease by 80% [30]. In general, the PAHs level in the sediment of Langkawi Island is low to moderate compared with the findings from previous studies. 

The dominant PAH compounds found in the sediment samples include naphthalene from two-ring PAHs (208 ng g^−1^) and chrysene from four-ring PAHs (178 ng g^−1^), with a content percentage of 18% and 15% of the total PAHs in all stations, respectively. Compared to the SQGs for ERL and ERM the amount of naphthalene was higher than the ERL (160 ng g^−1^) and lower than the ERM (2100 ng g^−1^); these results indicate that the probability of a negative toxic effect is lower than 50%. The amount of chrysene was also lower than ERL (384 ng g^−1^), which suggests that the probability of a negative toxic effect is lower than 10%. The high abundance of naphthalene is probably caused by a fresh input of fuel due to boat activities related to tourism. The inefficiency of the two-stroke outboard engines of most Langkawi boats causes them to discharge unburned fuel directly to the water column; this could be the reason for the high concentration of LMW-PAHs, especially naphthalene [39]. This finding corresponds with the findings of Tam et al. [4], who found a high level of naphthalene in the sediment of mangrove swamps in Hong Kong. Moreover, the abundance of chrysene is due to their very low solubility in water and high resistance to degradation. Wang et al. [40] arrived at the same conclusion when the level of chrysene in the sediment was not degraded even 12 years after an oil spill. 

Benzo[ghi]perylene (BgP), a compound with the fingerprint of a combustion engine and is abundant in soot [6], was found in sediment samples from the island. This compound has the fourth highest mean concentration for individual PAH (74.4 ng g^−1^), which is lower than the value indicated in the SQGs-ERL (85 ng g^−1^). The most probable source of BgP is the burning of fossil fuels (e.g., gasoline and diesel) of boats and vehicle engines commonly used in the island. A study by Omar et al. [41] also supported the emission of BgP from engines. The study mentioned that the highest abundance of BgP was recorded in the urban aerosols of Kuala Lumpur, Malaysia. The source of these aerosols was the incomplete fuel combustion.

Benzo[a]pyrene (BaP) is considered as the most hazardous of the seven carcinogenic PAHs [42]. An effective marker of pollution by PAHs was detected in all sediment samples from the jetties and fish farms, where the concentrations ranged from 18 to 55 ng g^−1^ (mean of 36.4 ng g^−1^), which are lower than the ERL and ERM SQGs of 430 ng g^−1^ and 1600 ng g^−1^, respectively. These results show that organisms, especially fish in these locations, are in safe condition at the island.

The relationship between the PAH concentration and the physicochemical characteristics of the sediment samples was analyzed. The results show no significant and negative correlation between the concentration of total PAHs and the concentrations of TOC and OM (*r* = −0.58, *r* = −0.35). These poor correlations are probably due to the differences in inputs of PAHs, TOC, and OM. [43]. Simpson et al. [44] suggested that the strong correlation between total PAHs and TOC is mostly significant for highly contaminated sites when the total PAH concentration is greater than 2000 ng g^−1^, which can explain the weak correlations in this study. These results are in agreement with the results of other studies such as Zhu et al. [43] and Ouyang et al. [45].

### 3.3. PAH Composition

**Figure 2:**
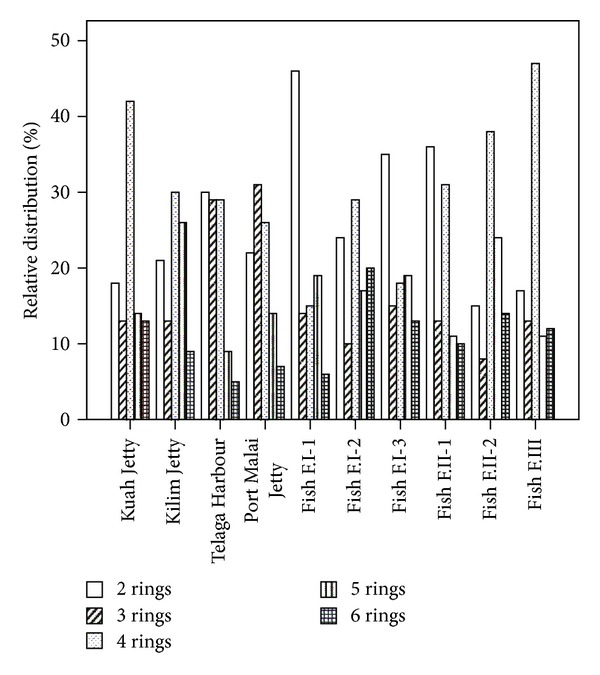
Relative distribution (%) of 2-, 3-, 4-, 5-, and 6-ring PAHs in the sediment samples of Langkawi Island.

**Figure 3:**
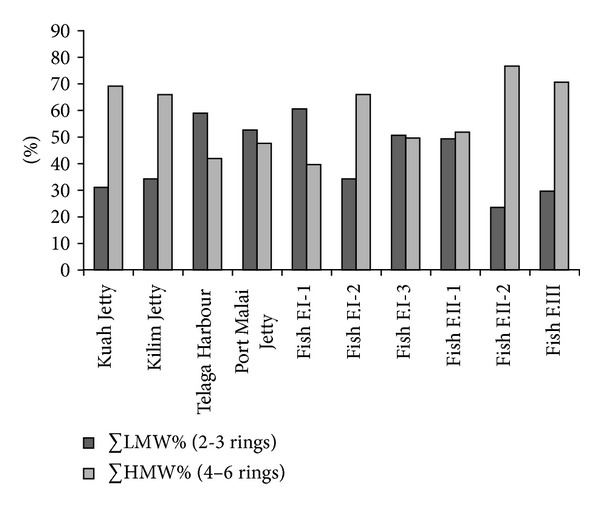
Relative percentages (%) of ∑LMW and ∑HMW in the sediment of different sampling stations.

The composition pattern of PAHs by ring size in the sediment samples around Langkawi Island is shown in Figure 2. On average, the high molecular weight PAHs with four rings (FIu, Pyr, BaA, and Chr), five rings (BbF, BkF, BaP, and DBA), and six rings (InP, BgP) account for 31%, 15%, and 11% of the total PAH concentrations, respectively. However, the lower molecular weight PAHs with two rings (1MNap, 2MNap, and Nap) and three rings (Acy, Ace, Fl, Phe, Ant) comprised 26% and 17% of the total PAH concentrations in the sediment, respectively. Sediment samples from the Kilim and Kuah jetties and fish farms I-1, II-2, and III were dominated by HMW-PAH (4 to 6 rings) (Figure 3) representing a range of 65.8% to 76.5%. The lower molecular weight LMW-PAHs (2 to 3 rings) were the most abundant components in the sediment sample of Telaga Harbour (59%) and fish farm I-1 (60.5%). Sediment sample from Porto Malai the fish farms I-3 and II-1 represented approximately an equal content of HMW-PAH and LMW-PAH (Figure 3). The results indicate that the high content of HMW fractions may be due to lower water solubility, less volatility, and higher persistence of the HMW compared with the LMW in an aquatic environment [46]. The major source of HMW-PAHs in this area is also probably anthropogenic activities [47] such as the incomplete fuel combustion of boats and vehicle engines as well as the unscrupulous disposal of engine oil from boats and ferries [30]. However, the high abundance of LMW-PAHs in some stations suggests relatively recent local PAH sources that entered the seawater [48], due to the inefficient two-stroke outboard engines of most boats in Langkawi Island. These engines usually discharge about 20% of unburned fuel directly into the water column [48]. The results of the paired sample *t*-test, which is used to display the difference between the means of two groups, of the LMW to HMW-PAHs show a significant difference between the means of LMW and HMW-PAHs in Langkawi Island sediment (*P* = 0.021), and the correlation coefficient between LMW and HMW-PAHs was insignificant and negative (*r* = −0.316), suggesting different inputs for both LMW and HMW PAHs in the sediment of Langkawi. 

### 3.4. Identification of PAH Sources

Diagnostic ratios and principal component analysis (PCA) are used to explain the details regarding the sources of PAHs sources in sediment samples [49–51].

#### 3.4.1. Diagnostic Ratios

Diagnostic ratios are used to distinguish the sources, petrogenic and pyrogenic, of PAH in different environment media depending on their physical and chemical properties and stability against photolysis [51]. Several PAH diagnostic ratios have been selected as indicators that have the most potential to distinguish between petrogenic and pyrogenic sources and are the most consistently quantifiable compounds in the majority of these samples. This includes the ratios of Ant/Ant+Phe, BaA/BaA+Chr, and LMW-PAH to HMW-PAH [9, 51, 52].  Table 6 shows the diagnostic ratios of several PAH compounds and their possible sources. The ratio of Ant/Ant+Phe of >0.1 indicates a dominance of heavy fuel combustion, whereas a ratio of <0.1 suggests petroleum sources [9]. In our study, the values of the Ant/Ant+Phe ratio were between 0.10 and 0.61 (mean: 0.34), which suggests that the PAHs are from a combustion source. A possible contribution source of PAH in the island is the fuel combustion of boats and vehicle engines that are transported to sea by direct dry and wet deposition from the atmosphere and rainwater runoff [53]. 

In addition, a BaA/BaA+Chr ratio of <0.2 usually implies a petrogenic origin, 0.2 to 0.35 indicates a mixed petrogenic and pyrogenic origin, and >0.35 indicates pyrogenic origin [51]. The values of this ratio from the sediment samples ranged from 0.06 to 0.74 (mean = 0.34) (Table 6). Kuah Jetty, fish farm I, and Fish farm III showed petrogenic inputs, while the Kilim and Proto Malai jetties, Telaga Harbour, fish farm I-1, and fish farm I-3 indicate a strong pyrogenic origin. Fish farm II-2 represented a mixture of petrogenic and pyrogenic inputs, which may might come from direct discharge of two-stroke engine boats and the deposition of fuel combustion from boats and vehicles. The LMW/HMW ratio was relatively low for most sites, ≤1, suggesting a pyrogenic origin of PAHs at these sites 0.31–1.53. 

However, these ratios generally suggest that PAHs can be largely attributed to the fuel combustion of petrogenic origin. Distinguishing the sources of PAH depends on the chosen diagnostic ratios, which can reveal pyrogenic material inputs. Moreover, the decrease in HMW-PAHs compared to the LMW-PAHs in some stations can also reflect a lesser contribution by petrogenic sources due to the direct discharge of unburned fuel from two-stroke engine boats. 

#### 3.4.2. Principal Component Analysis

PCA is a statistical tool that resets large data and allows the easy visualisation of similarities and differences between data sets [49]. PCA results were characterised by five principal components (PC1 to PC5) accounting for 21.8%, 19.5%, 17.4%, 11.8%, and 11.7% of the total variance, respectively (Table 7). Loading scores higher than 0.3 are considered meaningful. PC1 has significant positive loadings for fluorene, anthracene, chrysene, pyrene, and benzo[ghi]perylene, which have pyrogenic fingerprint. These are usually the result of the complete and incomplete combustion of petroleum products. PC2 has loads of alkylated naphthalene, acenaphthylene, acenaphthene, fluoranthene, and benz[a]anthracene. Based on this composition, PC2 is essentially the petrogenic components with low-temperature pyrogenic sources such as boats and ships engines. PC3 is predominantly composed of benzo[b]fluoranthene, benzo[k]fluoranthene, and benzo[a]pyrene, which are similar to the PAH compositions of engine emission [54]. PC4 had significant positive loadings for indeno[1,2,3-cd]pyrene and dibenzo[ah]anthracene, which could be attributed to pyrogenic sources such as gasoline engines, lubrication oil, and used motor oils. PC5 had positive loadings for phenanthrene and naphthalene, which reflected petrogenic components that could be attributed to the direct discharge of two-stroke boat engines. The results of the PAH source distribution indicated that the major sources of all PAHs were petrogenic inputs, such as the direct discharge of two-stroke engine boats, and pyrogenic inputs, such as the deposition of complete and burning of fossil fuel from boats, ferries, ships, and vehicles.

### 3.5. Sediment Potential Toxicity Based on Carcinogenic PAHs (CPAHs)

The toxicity assessment of Langkawi sediment was carried out according to the total concentration of seven potentially carcinogenic PAHs, including BaA, Chr, BbF, BkF, BaP, DBA, and InP [12]. The sum concentrations of the seven CPAHs ranged from 270.4 to 744.3 ng g^−1^ d.w., with a mean concentration of 475.1 ± 63.5 ng g^−1^ d.w. representing 27.5 to 63.3% of the total PAHs in the sediment of Langkawi Island. These results are lower than the SQGs of CPAHs, an ERL of 1373 ng g^−1^, and an ERM of 8410 ng g^−1^ [10]. 

**Figure 4:**
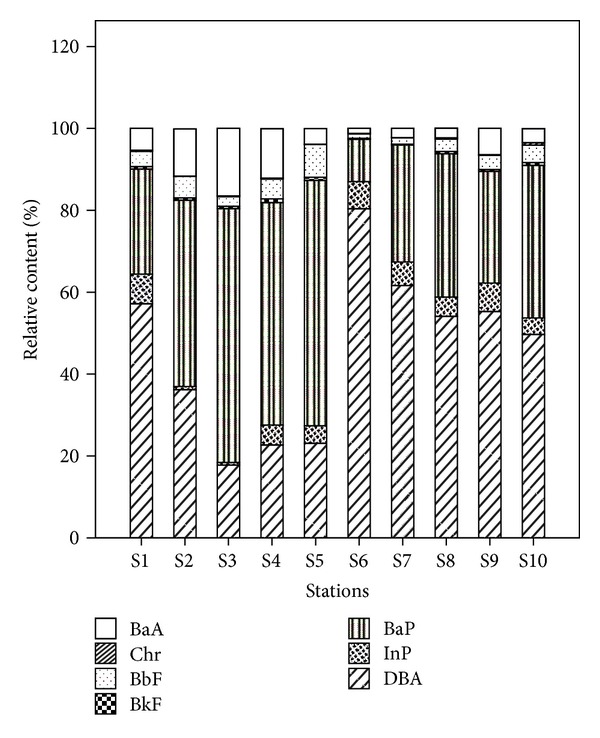
Relative contents of toxic BaP of potentially carcinogenic PAHs in sediments from the ten stations of Langkawi Island.

Among all known potentially carcinogenic PAHs, BaP is the only PAH for which toxicological data are sufficient to derive a carcinogenic potency factor [54]. The potential toxicity of sediment was assessed by calculating the total toxic BaP equivalent (TEQ carc) for all carcinogenic PAHs using the following equation [12, 55]:

(1) $$\begin{array}{c} \text{Total}\;\text{TEQ}\;\text{carc}=\sum_{i}C_{i}\times \text{TEF}i\;\text{carc}, \end{array}$$

where *C*_*i*_ is the concentration of individual carcinogenic PAH (ng g^−1^ d.w.) and TEF*i* carc (toxic equivalency factors) is the toxic factor of carcinogenic PAHs relative to BaP. The US Environmental Protection Agency [56] established the TEFs for each CPAHs: 0.1 for BaA, 0.001 for Chr, 0.1 for BbF, 0.01 for BkF, 1 for BaP, 0.1 for IP, and 1 for DBA. Total TEQ carc calculated for all samples investigated in this study ranged from 76.3 to 174.6 ng TEQ g^−1^ d.w., with a mean concentration of 107 ± 24 ng TEQ g^−1^ d.w. (Table 8, Figure 4). The probable sources of these compounds were the burning of fossil fuels of the boats, ships and vehicle engines extensively used in the island. In comparison with the published studies, TEQ carc values were lower in the sediment of Langkawi Island than those of other areas reported in other studies, such as the sediment from the Barents Sea in Guba pechenga, Russia [55], Meiliang Bay in Taihu Lake, China [13], and Kaohsiung Harbor in Taiwan [12]. The contribution of each carcinogenic PAH and the average values of relative contents, to the total TEQ carc varied according to the following order: DBA (45.8%), BaP (38.6%), BaA (6.5%), InP (4.6%), BbF (3.8%), BkF (0.5%), and Chr (0.2%).

### 3.6. Potential Ecosystem Risk Assessment

To assess the potential toxicity influence of Langkawi Island sediment on the surrounding sea organisms and their ecosystem, PAH levels in the sediment of the island were compared with the sediment toxicity screening guideline of the US National Oceanic and Atmospheric Administration, which include two target values: ERL and ERM [10]. 

Table 9 shows the concentration ranges of individual PAH for all stations. Some have concentrations lower than the ERL values and others have concentrations higher than the ERL. As shown in Table 9, all individual PAH concentrations were below the ERM, which indicates that no high negative toxic effect can occur in this area. However, several individual PAHs in some sites were above ERL and below ERM. Which were occasionally caused negative toxic effects with a range of 10–50% on the surrounding sea organisms and their ecosystem. For example, at station S1 (Ace), S2 and S5 (Nap, Ace), S3 (Nap, 1MNap, Acy, Ace), S4 (Nap, Acy, Ace), S6 (Nap, Ace, Fl, DBA, BgP), S7 (Nap, Ace, DBA), S8 (Ace), S9 (DBA), and S10 (Ace, Fl, Chr, BgP).

In addition, another approach that can be used to evaluate the possible biological effects or toxicity of PAHs in sediment is the mean ERM quotient (m-ERM-q). This approach calculates the mean quotients for all PAHs according to the formula suggested by Long and MacDonald [57]: m-ERM-q = ∑(*C*_*i*_/ERM*i*)/*n*, where *C*_*i*_ is the concentration of PAH, ERM*i* is the ERM value for the same target of PAH, and *n* is the number of PAH. As mentioned in Long et al. [58], m-ERM-q can be categorised into four levels according to their probability of toxicity: ≤0.1 indicates an 11% probability of toxicity; 0.11 to 0.5 indicates a 30% probability of toxicity; 0.51 to 1.5 indicates a 46% probability of toxicity, and >1.5 indicates a 75% probability of toxicity. Moreover, the probability percentage of toxicity in these four categories can be used to classify the sampling sites as low, medium-low, medium-high, and high-priority sites, respectively. According to these categories and classifications, the m-ERM-q values of the sediment from each site in Langkawi Island ranged from 0.04 to 0.10 with a mean value of 0.07. The results of this study can be ranked under the first category, where the value is less than 0.1 with an 11% probability of toxicity. They are also classified as low-priority sites.

## 4. Conclusion

PAHs were detected in all surface sediment samples collected from four jetties and three marine fish farms around the main Langkawi Island. Concentrations of total PAHs varied from 869 to 1637 ng g^−1^ d.w. with a mean concentration of 1167 ng g^−1^ d.w. Concentrations did not exceed the SQG ERL (3442 ng g^−1^), indicating the absence of acute biological effects. The possible source of PAHs in the majority of sediment samples from Langkawi Island is pyrogenic such as from incomplete and complete petroleum combustion from boats, ships, and vehicle engines. In other areas, the sources could be petrogenic such as from nonburnt fuel discharge of two-stroke engine boats. The results of potential toxicity and biological effect assessment show that the surface sediments from Langkawi Island have low contamination and a low probability of toxic pollution.

## Figures and Tables

**Table 1:** Sampling locations and their associated water depths.

Number	Sampling station	Station name	Depth (meter)	Latitude (N)	Longitude (E)
1	S1	Kuah Jetty	3	06° 18′ 22.9′′	099° 51′ 02.0′′
2	S2	Kilim Jetty	1	06° 24′ 18.4′′	099° 51′ 31.0′′
3	S3	Telaga Harbour	3	06° 22′ 03.6′′	099° 41′ 07.0′′
4	S4	Porto Malai Jetty	3	06° 15′ 57.5′′	099° 44′ 13.3′′
5	S5	Fish farm I-1	11	06° 13′ 42.9′′	099° 46′ 47.8′′
6	S6	Fish farm I-2	11	06° 13′ 52.9′′	099° 45′ 40.8′′
7	S7	Fish farm I-3	11	06° 14′ 12.9′′	099° 47′ 07.8′′
8	S8	Fish farm II-1	10	06° 12′ 48.0′′	099°.45′ 32.5′′
9	S9	Fish farm II-2	10	06° 12′ 48.0′′	099°.45′ 42.5′′
10	S10	Fish farm III	11	06° 16′ 39.6′′	099° 48′ 15.2′′

**Table 2:** Average recovery of 18 PAHs in marine sediment (SRM 1941b).

PAHs	Abbreviation	Recovery% ± SDV
Naphthalene	Nap	123 ± 14
1-Methylnaphthalene	1MNap	137 ± 13
2-Methylnaphthalene	2MNap	74 ± 6
Acenaphthylene	Acy	133 ± 11
Acenaphthene	Ace	128 ± 3
Fluorene	Fl	99 ± 4
Phenanthrene	Phe	94 ± 2
Anthracene	Ant	71 ± 11
Fluoranthene	FIu	73 ± 10
Pyrene	Pyr	79 ± 4
Benzo[a]anthracene	BaA	74 ± 8
Chrysene	Chr	87 ± 24
Benzo[b]fluoranthene	BbF	99 ± 16
Benzo[k]fluoranthene	BkF	77 ± 15
Benzo[a]pyrene	BaP	79 ± 20
Indeno[123-cd]pyrene	InP	75 ± 11
Dibenzo[ah]anthracene	DBA	106 ± 20
Benzo[ghi]perylene	BgP	90 ± 32

**Table 3:** TOC, OM, and grain size of sediments from Langkawi Island (values are in percentage).

Stations	OM^a^	TOC^b^	Gravel^c ^	Sand^c^	Silt + Clay^c^
Kuah Jetty	11.94	1.50	5.35	93.87	—
Kilim Jetty	19.67	3.17	1.29	98.10	—
Telaga Harbour	11.48	0.66	9.94	90.01	0.15
Porto Malai Jetty	10.26	1.27	9.22	89.72	0.33
Fish farm I-1	16.90	1.13	5.66	93.22	1.02
Fish farm I-2	16.82	0.95	2.85	96.22	0.18
Fish farm I-3	21.56	1.41	—	99.41	0.21
Fish farm II-1	22.41	1.36	—	98.90	0.50
Fish farm II-2	21.39	1.33	0.22	99.09	0.14
Fish farm III	20.49	1.24	—	99.75	0.16

^a^OM: organic matter; ^b^TOC: total organic carbon;^ c^classification of grain size sediment.

**Table 4:** Concentrations of 18 PAHs in sediment of Langkawi Island (ng g^−1^) (mean ± SD).

PAH	Kuah Jetty	Kilim Jetty	Telaga Harbour	Porto Malai Jetty	Fish F.I-1	Fish F.I-2	Fish F.I-3	Fish F.II-1	Fish F.II-2	Fish F.III
Nap	72 ± 16	169 ± 66	325 ± 3	164 ± 59	362 ± 141	164 ± 20	293 ± 42	269 ± 140	110 ± 39	151 ± 99
1MNap	65 ± 0	12 ± 4	94 ± 10	65 ± 5	63 ± 42	44 ± 18	19 ± 10	81 ± 44	31 ± 10	47 ± 20
2MNap	53 ± 0	2 ± 1	70 ± 20	32 ± 9	26 ± 17	86 ± 36	13 ± 3	49 ± 6	37 ± 4	44 ± 19
Acy	16 ± 19	21 ± 18	159 ± 26	66 ± 10	16 ± 16	36 ± 22	13 ± 3	39 ± 0	35 ± 20	38 ± 9
Ace	20 ± 7	30 ± 5	242 ± 154	259 ± 121	17 ± 4	17 ± 7	31 ± 9	32 ± 9	15 ± 13	26 ± 14
Fl	8 ± 5	6 ± 1	12 ± 3	10 ± 3	16 ± 17	21 ± 23	4 ± 0	8 ± 4	3 ± 3	37 ± 45
Phe	39 ± 5	40 ± 7	52 ± 3	19 ± 1	65 ± 23	25 ± 3	64 ± 5	64 ± 15	39 ± 6	44 ± 2
Ant	60 ± 14	17 ± 13	10 ± 3	23 ± 1	24 ± 1	27 ± 12	29 ± 10	7 ± 0	7 ± 7	36 ± 37
FIu	54 ± 15	33 ± 6	79 ± 2	40 ± 13	36 ± 11	23 ± 1	35 ± 1	391	41 ± 21	33 ± 6
Pyr	94 ± 1	43 ± 9	81 ± 21	71 ± 7	57 ± 3	82 ± 31	65 ± 3	73 ± 1	66 ± 32	162 ± 88
BaA	44 ± 1	139 ± 7	145 ± 31	94 ± 16	29 ± 12	21 ± 3	30 ± 2	17 ± 1	112 ± 16	28 ± 14
Chr	260 ± 12	49 ± 35	162 ± 17	116 ± 13	26 ± 10	231 ± 70	33 ± 15	218 ± 23	230 ± 117	455 ± 247
BbF	31 ± 8	63 ± 15	21 ± 16	38 ± 3	61 ± 55	18 ± 7	21 ± 21	21 ± 0	60 ± 40	34 ± 17
BkF	49 ± 3	64 ± 24	49 ± 10	68 ± 43	58 ± 60	39 ± 36	31 ± 21	40 ± 0	76 ± 43	53 ± 11
BaP	21 ± 1	55 ± 16	43 ± 23	42 ± 32	46 ± 15	18 ± 3	38 ± 6	24 ± 1	48 ± 36	29 ± 11
InP	60 ± 4	9 ± 5	5 ± 8	38 ± 0	32 ± 4	112 ± 58	77 ± 55	32 ± 7	122 ± 19	32 ± 1
DBA	47 ± 1	43 ± 11	16 ± 7	18 ± 5	18 ± 12	136 ± 39	82 ± 74	38 ± 7	97 ± 53	39 ± 11
BgP	82 ± 0	73 ± 25	72 ± 20	52 ± 36	22 ± 5	131 ± 68	44 ± 21	77 ± 10	48 ± 32	143 ± 86

∑18PAHs	1074 ± 19	868 ± 85	1637 ± 190	1213 ± 248	974 ± 96	1231 ± 376	922 ± 170	1129 ± 192	1176 ± 242	1432 ± 693

**Table 5:** PAH concentrations (ng g^−1 ^d.w.) in sediments from various marine sites in the world.

Locations	*N* ^ a^	Concentrations	Reference
Langat Estuary-Malaysia	17	322–2480	[46]
East coast of Malaysia	17	260–590	[59]
Singapore Island	15	15220–82410	[31]
Egypt-Mediterranean sea	39	13.5–22,600	[16]
Naples harbour, southern Italy	16	9–31774	[32]
Gulf of Fos area, France, Mediterranean sea	13	34–2700	[60]
Tokyo Bay, Japan	26	1372–1615	[33]
Italian marine protected areas (MPA)	16	0.71–1550	[61]
Jakarta Bay, Indonesia	26	257–1511	[33]
Jiulong River Estuary and Western Xiamen Sea, China	16	59–1177	[34]
Marine sediments in Thailand	15	6–228	[35]
Estero de Urias Estuary, Mexico	12	27–418	[36]
Italy, Mediterranean sea	16	40–679	[62]
Gulf of Aden, Yemen	46	2.2–604	[37]
Southwest Taiwan	28	15–907	[38]
Hong Kong (fish farms)	16	123–947	[26]
Langkawi Island, Malaysia	18	868–1637	Present study

^a^Number of PAHs.

**Table 6:** Diagnostic PAH ratios in the sediment and their possible sources.

	BaA/BaA + Chry^a^		An/An + Ph^b^		LMW/HMW^c^	
Kuah Jetty	0.14	Petr	0.61	Pyr	0.45	Pyr
Kilim Jetty	0.74	Pyr	0.29	Pyr	0.52	Pyr
Telaga Harbour	0.47	Pyr	0.16	Pyr	1.43	Pyr
Porto Malai Jetty	0.45	Pyr	0.55	Pyr	1.11	Pyr
Fish F.I-1	0.53	Pyr	0.27	Pyr	1.53	Pyr
Fish F.I-2	0.08	Petr	0.52	Pyr	0.52	Pyr
Fish F.I-3	0.47	Pyr	0.31	Pyr	1.02	Pyr
Fish F.II-1	0.07	Petr	0.10	Pyr	0.93	Pyr
Fish F.II-2	0.33	Petr + Pyr	0.15	Pyr	0.31	Pyr
Fish F.III	0.06	Petr	0.45	Pyr	0.42	Pyr
Mean	0.34		0.34		0.82	
Petrogenic S.	<0.2		<0.1		>1	
Pyrogenic S.	>0.35		>0.1		<1	

^a^Benzo[a]anthracene to benzo[a]anthracene plus chrysene; ^b^anthracene to anthracene plus phenanthrene ratio; ^c^PAHs with low molecular weight to PAHs with high molecular weight; Petr: petrogenic; Pyr: pyrogenic.

**Table 7:** Rotated component loadings of the principal components (PCs) for PAH composition in the sediment of Langkawi Island.

PAHs	PC1	PC2	PC3	PC4	PC5
Naphthalene	—		—	—	0.87
1-Methylnaphthalene	—	0.55	—	—	0.35
2-Methylnaphthalene	0.33	0.49	—	0.49	—
Acenaphthylene	—	0.97	—	—	—
Acenaphthene	—	0.82	—	—	—
Fluorene	0.87	—	—	—	—
Phenanthrene		—	—	—	0.90
Anthracene	0.62	—	—	—	—
Fluoranthene	—	0.77	—	—	—
Pyrene	0.96		—	—	—
Benzo[a]anthracene	—	0.61	0.39	—	—
Chrysene	0.89	—	—	—	—
Benzo[b]fluoranthene	—	—	0.91	—	—
Benzo[k]fluoranthene	—	—	0.86	—	—
Benzo[a]pyrene	—	—	0.90	—	—
Indeno[123-cd]pyrene	—	—	—	0.87	—
Dibenzo[ah]anthracene	—	—	—	0.86	—
Benzo[ghi]perylene	0.86	—	—		—
Explained variance (%)	21.8	19.5	17.4	11.8	11.7

Rotation method: Varimax with Kaiser Normalization.

—: PCA loading values lower than 0.3 are not presented.

**Table 8:** Concentration of carcinogenic PAHs (ng g^−1^ dry wt.) and total toxic BaP equivalent (total TEQ, ngTEQ g^−1 ^dry wt.) in sediments from different locations around the world; data show range.

Location	*n*	∑CPAHs	∑TEQ carc	Reference
Barents Sea, Russia	7	864–63	18–300	[55]
Kaohsiung Harbor, Taiwan	7	256–8067	55–1964	[12]
Meiliang Bay, China	5	621–2737	94–856	[13]
Sediment quality guidelines (ERL)	7	1373	—	[10]
Sediment quality guidelines (ERM)	7	8410	—	[10]
Langkawi Island, Malaysia	7	270–744	76.3–174.6	Present study

**Table 9:** Concentration ranges of PAHs in sediment from Langkawi Island and toxicity guidelines.

	SQG, ng g^−1^		PAH concentration ng g^−1^d.w. (s)		Stations (S)			
	ERL	ERM	MIN	MAX	S < ERL	ERL < S < ERM	Name of S < ERM	S OF > ERM
Nap	160	2100	72	362	3	7	S2, S3, S4, S5, S6, S7, S8	—
1MNap	85	800	12	94	9	1	S3	—
2MNap	70	670	2	86	10	—		—
Acy	44	640	13	159	8	2	S3, S4	—
Ace	16	500	15	259	1	9	S1, S2, S3, S4, S5, S6, S7, S8, S10	—
Fl	19	540	3	21	8	2	S6, S10	—
Phe	240	1500	19	65	10	—		—
Ant	85	1100	7	60	10	—		—
FIu	600	5100	23	79	10	—		—
Pyr	665	2600	43	162	10	—		—
BaA	260	1600	17	145	10	—		—
Chr	380	2800	26	455	9	1	S10	—
BbF	—	—	18	63	—	—		—
BkF	—	—	31	76	—	—		—
BaP	430	2800	18	55	10	—		—
InP	240	950	5	112	10	—		—
DBA	63	260	16	136	7	3	S6, S7, S9	—
BgP	85	330	22	143	8	2	S6, S10	—

∑PAHs	3442	24290	362	2532				

SQGs: sediment quality guidelines, ERL: effect range-low, ERM: effect range-median.
